# Te Ira Tangata: A Zelen randomised controlled trial of a treatment package including problem solving therapy compared to treatment as usual in Maori who present to hospital after self harm

**DOI:** 10.1186/1745-6215-12-117

**Published:** 2011-05-11

**Authors:** Simon Hatcher, Nicole Coupe, Mason Durie, Hinemoa Elder, Rees Tapsell, Karen Wikiriwhi, Varsha Parag

**Affiliations:** 1 Department of Psychological Medicine, Faculty of Medical and Health Sciences, The University of Auckland, Private bag 92019, Auckland, New Zealand; 2 Deputy Vice Chancellor (Māori) & Professor Māori Research & Development Massey University, Private Bag 11-222, Palmerston North, New Zealand; 3 Hauora Waikato, 474 Anglesea St, PO Box 1283, Hamilton, New Zealand; 4 Psylaw, PO Box 44338, Pt Chevalier, Auckland, New Zealand; 5 Clinical Trials Research Unit, School of Population Health, The University of Auckland, New Zealand

## Abstract

**Background:**

Maori, the indigenous people of New Zealand, who present to hospital after intentionally harming themselves, do so at a higher rate than non-Maori. There have been no previous treatment trials in Maori who self harm and previous reviews of interventions in other populations have been inconclusive as existing trials have been under powered and done on unrepresentative populations. These reviews have however indicated that problem solving therapy and sending regular postcards after the self harm attempt may be an effective treatment. There is also a small literature on sense of belonging in self harm and the importance of culture. This protocol describes a pragmatic trial of a package of measures which include problem solving therapy, postcards, patient support, cultural assessment, improved access to primary care and a risk management strategy in Maori who present to hospital after self harm using a novel design.

**Methods:**

We propose to use a double consent Zelen design where participants are randomised prior to giving consent to enrol a representative cohort of patients. The main outcome will be the number of Maori scoring below nine on the Beck Hopelessness Scale. Secondary outcomes will be hospital repetition at one year; self reported self harm; anxiety; depression; quality of life; social function; and hospital use at three months and one year.

**Discussion:**

A strength of the study is that it is a pragmatic trial which aims to recruit Maori using a Maori clinical team and protocol. It does not exclude people if English is not their first language. A potential limitation is the analysis of the results which is complex and may underestimate any effect if a large number of people refuse their consent in the group randomised to problem solving therapy as they will effectively cross over to the treatment as usual group. This study is the first randomised control trial to explicitly use cultural assessment and management.

**Trial registration:**

Australia and New Zealand Clinical Trials Register (ANZCTR): ACTRN12609000952246

## Background

Maori are the indigenous people of New Zealand and make up about 15% of the population. Māori have a one year prevalence rate of "suicide attempts" that is three times higher than non-Mäori (0.9% twelve month prevalence compared to 0.3% in non-Maori) [1] and have a suicide rate that is about 30% higher than non-Maori (13.3/100,000 compared to 10.6/100,000)[2]. The challenge is to provide effective treatment for Maori who present to hospital with self harm that is culturally acceptable and meets the obligations of the Treaty of Waitangi.

There are over 5000 hospitalisations for self harm each year in New Zealand and a history of self harm is the most powerful predictor of subsequent suicide with about 1% of people going on to kill themselves in the year after a self harm attempt [3]. In the most recently updated Cochrane review of treatments for self harm no conclusive evidence was found for the efficacy of any single intervention in reducing repetition rates of self harm [4]. Subsequent to this review several large trials have reported their results with mixed findings. We propose a novel approach to this problem by providing a "package of care" delivered by specialised self harm teams. The package is an integrated combination of six interventions which are likely to make a difference to outcomes after self harm and address a variety of risk factors. This "package of interventions" has the potential to add to worldwide knowledge about the efficacy of interventions for self harm. The rationale for the interventions we have chosen to offer as part of Te Ira Tangata are described below.

Firstly there are postcards sent to patients after the episode of self harm. In 2005 Greg Carter developed an intervention in which a series of eight "postcards" were sent in sealed envelopes over one year after discharge to patients who had presented at emergency departments for self-poisoning [5]. At one year follow-up patients in the intervention group had half the number of readmissions than the control group (101 vs 192) although the proportion of people re-presenting in each group was not significantly different. In a more recent trial in Christchurch by Annette Beautrais [6] of a similar intervention there was no difference in outcomes once the history of self harm had been taken into account in both groups. However the study sample was small and included people presenting to a crisis team with suicidal ideas as well as presentations with self harm to the emergency department.

Secondly there is Problem-solving therapy (PST) which is a brief focused psychological treatment that has been shown to be significantly more effective than control conditions with regard to improvement in depression, hopelessness and problems in patients who have attempted suicide [7]. Evidence that problem solving therapy is effective in reducing repetition rates is less conclusive, although promising trends have been reported [8]. There is general agreement that problem solving therapy is a cost-effective, brief intervention that has the potential to be a feasible and effective addition to existing services [7].

Next is the issue of the high mortality rate from non suicide causes after self harm. About 50% of the premature mortality after self harm is due to non suicide deaths [9] and the overall rate of death may reach 15% five years after the index episode of self harm [10]. This suggests that treating self harm as purely a mental health problem will not address a key outcome.

A related problem is the issue of patients not getting the treatment that clinicians say they should receive in the management plans made in the emergency department. There are a variety of reasons for this including patients' resistance to attend appointments, referrals not being made in a timely fashion or contact details recorded in the emergency department being incorrect. There is some evidence that more intensive outreach after self harm results in better attendance in out-patients although it is unclear whether this decreases the repetition rate [8].

A difficult and controversial area is the management of risk for the individual and the team. Traditionally risk assessment in mental health, especially in the area of suicide prevention, has focused on prediction, using risk factors associated with the patient, so that patients are said to be at low, medium and high risk. The difficulty with this is that there is no evidence that clinicians can predict who will commit suicide and most people who kill themselves are low risk and most people who are high risk don't kill themselves [11]. This suggests that using risk assessment to predict who will kill themselves is flawed and that a better system is needed.

Lastly a neglected component of assessment in mental health, with the notable exception of 'cultural services', is the clinical assessment of identity and belonging in people who self harm. This is particularly surprising given that firstly having a damaged autobiographical memory [12] and a poor sense of belonging [13] are relatively common in people who self harm and secondly that there is a tradition within psychiatry that sees development of a strong sense of identity as desirable for good mental health. Kaupapa Maori services have of course seen the development of a secure identity as an essential part of their task. Also those who receive problem solving will be prompted to consider "not belonging" as a potential problem to be addressed. Previous research has shown that having a secure cultural identity is less common in Māori who self harm compared to a control population [14]. The proposed study follows on from this research and is an attempt to address issues of cultural identity in Māori who self harm to see if this protects them from further self harm and improves other outcomes.

We propose to include each of these components in a package of care delivered to individuals after they present with self harm. By combining them together we aim to replicate the package of interventions that a clinical "self harm team" could reasonably deliver and to test the idea that the effect of the package is more than the sum of the individual parts. The parts of the package will be sending postcards over a year; the offer of brief problem solving therapy; patient support which will be a mainly telephone based system of case management to "stop people falling through the cracks" after their presentation to hospital; vouchers that will allow patients to access their GP's for free with an emphasis on physical health checks and ensuring registration with a GP; a systemic approach to identifying and managing modifiable risk factors in the patient and the self harm team based on the principles successfully used in managing risk in aviation; and a cultural assessment focused on sense of belonging.

The package of care that will be delivered as part of Te Ira Tangata will meet the three principles that underpin good clinical practice from a Maori perspective [15]. These are:

I. The principles of indigeneity which recognises indigenous peoples rights to exercise a degree of autonomy. The face to face problem solving in Te Ira Tangata will be developed by Maori to ensure that the "rituals of encounter", the delivery and content are acceptable to Maori.

II. The principle of clinical expertise which acknowledges the rights of Mäori to get the best treatment available. The package of care delivered in this study combines the evidence based treatments which are most likely to improve outcomes for Mäori who present to hospital with self harm.

III. The principle of cultural competence which focuses on clinicians ability to be competent to work in their own and others cultures. In Te Ira Tangata the research clinicians are Maori seeing Maori patients supported by Mäori cultural workers.

This study begins to address the need for an evidence base for the effectiveness of Maori specific services that is clearly articulated by Tapsell in his description of similar services in a forensic psychiatry setting [16].

### Why use a Zelen design?

Systematic reviews have identified small randomised trials of unrepresentative patients as a problem in this area. This study uses a double consent Zelen randomisation design [17]. In this design individuals are randomised before they give consent. The reason for choosing this design, rather than the standard randomised controlled trial design, is that in a standard randomised controlled trial clients are required to understand complex concepts such as randomisation and clinical equipoise before giving consent. Such an approach is likely to be inappropriate for people in crisis, in an emergency room, and who are often physically unwell following a self harm episode. Consequently the use of a Zelen design has the potential to improve recruitment rates as the conversation with eligible patients is simpler. Also the people who consent to randomisation in conventional trials may well be unrepresentative of the people who present following self harm [8]. More importantly, if people are offered the possibility of receiving problem-solving therapy but then find that they have been randomised to receive treatment as usual only, this may result in higher rates of non-compliance in the control group as well as the possibility of "resentful demoralisation" [18], resulting in higher drop-out rates from the control group or reporting of lower scores on outcome measures.

The aim of the study is to see whether, in Māori who present to hospital after self harm, a culturally informed package of care, Te Ira Tangata, leads to better outcomes than treatment as usual alone. Specifically we have the following hypotheses:

1. A culturally informed treatment is more effective than treatment as usual in reducing the proportion of Maori who score below nine on the Beck Hopelessness Scale at one year. This is the primary outcome.

2. A culturally informed treatment is more effective than treatment as usual in reducing hospital representations with self harm, self-reported repetition of self-harm, hopelessness, depression, anxiety and health service use after three months and one year.

3. Māori who receive Te Ira Tangata are more likely to engage with support than those who receive current usual treatment in New Zealand.

4. The package of care plus treatment as usual will improve quality of life and function at three months and one year compared to treatment as usual.

5. The package of care will be more cost effective than treatment as usual.

## Methods

### Design

We will use a Zelen randomised controlled design to compare the package of care plus treatment as usual to treatment as usual in Maori who presented to hospital with self harm.

### Settings

The study will be conducted in four hospitals in three District Health Boards (DHB) in New Zealand - Waitemata DHB (North Shore Hospital and Waitakere Hospital), Counties Manukau DHB (Middlemore Hospital) and Northland DHB (Whangarei Hospital). Waitemata District Health Board provides health services for a population of about 525,000 people in urban north and west Auckland and a rural area north of the city with about 17% of its population living in the most deprived areas; Counties Manukau provides health care for 470,000 people in the South of Auckland and serves a population that is relatively young with a high proportion of Maori and recent immigrants and about a third of the population living in areas that are very deprived (http://www.cmdhb.org.nz/about_cmdhb/overview/population-profile.htm); Northland District Health Board serves a mainly rural area of about 150,000 characterised by a large Maori population, widely dispersed rural communities and a disproportionately high level of socio-economic deprivation.

### Participants

All adult Māori patients (that is those who are not still at secondary school) who present to the emergency departments of North Shore, Waitakere, Middlemore and Northland hospitals who are able to give informed consent will be eligible for the study. Māori patients will be approached by a research team member while in hospital or within 48 hours of discharge. Patients who require an interpreter will be included in the study. Self-harm is defined as intentional self-poisoning or self-injury, irrespective of motivation. Self-poisoning includes the intentional ingestion of more than the prescribed amount of any drug, whether or not there is evidence that the act was intended to result in death. This also includes poisoning with non-ingestible substances (for example pesticides or carpet cleaner), overdoses of 'recreational drugs' and severe alcohol intoxication where the clinical staff consider such cases to be an act of intentional self-harm. Self-injury was defined as any injury that has been intentionally self-inflicted [19]. We will assess the degree of suicidal intent by using a modified self report version of the Beck Suicide Intent Scale which we will use in the analysis to assess the impact suicidal intent on outcomes.

People will be excluded if they are aged under 17; are still at school; are unable to give informed consent to be part of the study, that is, if they are too mentally unwell (for example they are psychotic or hypomanic); if they are too physically unwell (for example, they are in a coma or with lowered level of consciousness); or if they are severely cognitively impaired.

### Recruitment

Following a psychosocial assessment by a non-study mental health clinician, patients will be handed a card by non-study clinician which informs them that they will be approached to participate in a study about what happens after self-harm (Additional file 1). If patients do not want to be contacted they will be asked to inform one of the non-study staff. Eligibility for the study will be assessed by a research therapist reviewing the notes. Eligible patients will be randomised and then approached by the research clinician to explain the study and to request consent to participate.

The maximum delay between the psychosocial assessment and the attempt to obtain consent is four days to allow for weekends and public holidays when research staff are not available. In practice we aim to approach potential participants within 24 hours of their presentation to the hospital. The approach will occur either within the Emergency Department, in hospital if the person is admitted, or where necessary, by telephone after the patient is discharged. An interpreter will be made available to any person who requests one.

### Randomisation and blinding

As this is a Zelen trial randomisation will be prior to obtaining consent. All eligible participants are allocated randomly to the intervention or usual care groups using a central computerised randomisation system at the Clinical Trials Research Unit (http://www.ctru.auckland.ac.nz). Stratified minimisation randomisation will be used to ensure a balance in key prognostic factors between the study groups: site (Waitemata DHB, Counties Manukau DHB, Northland DHB), history of self-harm (none, repeater), and method of self-harm (overdose, self injury, both). The assessors will be blind to the intervention group at the three and twelve months follow up assessments.

### The intervention

The content of the intervention meets the aims of Paiheretia as described by Durie [20]. That is we aim to help the client develop a secure identity, we pay attention to their relationships which may need healing and the problem solving encourages reciprocity with the wider environment. As well as the content we aim to make the process of therapy explicitly incorporate Maori cultural beliefs and values which we have chosen to describe using the powhiri model described below.

### The powhiri model

Each iwi (tribe) throughout Aotearoa (New Zealand) practises their own variation of kawa (protocol) on their home marae (tribal meeting places). With this research there will be 132 Māori presenting to three DHB regions (Northland, Waitemata and Counties Manukau) from a number of iwi, not necessarily mana whenua (people of that region). Therefore, a powhiri (welcoming ceremony) process is the most appropriate method of engagement when Māori researchers and Māori participants come together.

Before the powhiri, the kawa is conveyed to the visitors so that they understand what is expected of them. Thus the kawa determines how both the hosts and visitors interact within the sacred boundaries of the marae. For the purposes of this research the marae may equate to the clinical setting, hauora, Māori organisation or local marae.

The conveying of the kawa for Te Ira Tangata is the first level of engagement and can be indicated through the sharing of information through a brief conversation and the information sheets.

#### Taki/Wero - Challenge

Before the powhiri can begin, the tangata whenua (researchers) must challenge the manuhiri (visitors - participants) to make sure of their intentions and ensure they have come in peace. If their intentions are friendly, the participants will accept the rautapu (a leaf or carved effigy) a symbolic offering of peace. Once the challenge is completed, the warriors will guide you on to the sacred ground of the marae, while the karanga or call of the women of the tribe welcomes you.

Te Ira Tangata will offer the rautapu in the form of a consent form if the participant accepts the peace offering they will sign and return the form to participate in the study.

#### Karanga - Call

Karanga refers to the ceremonial call of welcome. The start of the karanga indicates to a visitor that they are free to approach their hosts across the marae atea (sacred space directly in front of the meeting house). The call also clears a spiritual pathway for the ancestors of both visitor and host to meet and partake in the ceremonial uniqueness of the powhiri. The call acknowledges the ancestral spirits of the visitors before them. The karanga acknowledges who you are and why you have come, and invite you to stop and shed tears for those who have passed on.

This is the beginning of the 'Patient Support' process within Te Ira Tangata. Where the tangata whenua (research clinician and administrator) have already reviewed clinical notes, checked any management plans and whether they need updating, offered GP voucher, performed risk assessments, referrals in operations and any change of circumstances.

#### Karakia - Blessing

Karakia are prayers that acknowledge a spiritual presence. When applied to a specific realm or occasion, each karakia would identify and acknowledge gods, demi gods and lesser spiritual deities so that nothing untoward should befall those involved. During a powhiri, the tangata whenua (people of the land) and manuhiri (visitors) often participate in karakia. This unites everyone present in body and spirit, and blesses the occasion. Once the karakia is completed, things begin to lighten up. It's time for the mihi and whaikorero, which amongst other things, talk about issues of the day and the reason for gathering.

Karakia is karakia in Te Ira Tangata and will be performed in appropriate sense for both the research clinician and the participant.

#### Mihi/Whaikorero - Greetings

The next phase of the powhiri involves the mihi and whaikorero, formal greetings exchanged between host and visitor. This phase is a very formal part of the powhiri. The hosts consider each visitor as sacred, according them all the rights that their position demands. As a visitor, you are expected to act in a dignified manner, for Māori accept your physical presence as representing all your ancestors. It is considered rude to show disinterest during these proceedings or talk over someone delivering their mihi. The exchange between research clinician and participant during PST forms the Mihi/Whaikorero.

There is a saying 'Ko te whaikorero, te kai a te Rangatira - Oratory is the food of Chiefs'. Today, as it was in former times, the arts of whaikorero and mihi are greatly revered. A good speaker will have both visitors and hosts in the palm of his hand, laughing or crying. A good research clinician will also be able to represent these skills during Te Ira Tangata.

Problem solving therapy has five steps including problem orientation, problem identification, generating solutions, making an action plan and reviewing the progress. As part of the problem orientation there will be an acknowledgement of:

I. the land

II. the dead

III. the reason for the gathering

IV. the wharenui (big house)

V. the people present.

This can form the cultural assessment process investigating a person's cultural identity profile. Using these outcomes a person's identity profile and sense of belonging will inform one of the 'problems' to be solved within the therapy.

#### Waiata - Song

The singing of waiata or song usually follows each mihi and whaikorero (formal greetings exchanged between host and visitor). Today waiata are sung in many languages and for many different reasons. Visitors that sing of their homeland or in their native tongue are said to bestow their hosts with the voice and sound of their ancestors. This is considered a great gift and honour.

At the end of the first PST session there is an opportunity for the participant to identify problems (list), chose one problem to work on, define the problem and generate a solution. When they leave the session they will be able to work on this and bring back progress to the next session.

#### Koha - Gift

Koha is the traditional act of gifting. In the powhiri (welcoming ceremony) the presentation of koha follows directly after the last speaker has finished their mihi (formal greetings) and waiata (song). The gifting of koha is a very dignified act.

In appreciation for participant contributions a gift will be given on the completion of the rating scales at baseline, 3 month follow up and 12 month follow up. This is a mark of reciprocity from the study to the participant.

#### Hongi - Coming together

The next stage of the powhiri, the hongi (pressing of noses), the ha or breath of life is exchanged and intermingled. Through the exchange of this physical greeting, you are no longer considered manuhiri (visitor) but rather tangata whenua, one of the people of the land. The hongi is also a sign of life symbolising the action of Tane's breath of life to humans. By this action the life force is permanently established and the spiritual and physical bodies become a living entity.

This symbolises the end of the therapy, participants have had their mauri (life force) restored, rejuvenated and supported.

#### Hakari - Feast

Hakari is the act of ritual feasting that traditionally applied to the eating of cooked food. Symbolically, the hakari recognises the transition from the spiritual realm of the powhiri back into the physical world where food is shared.

At the completion of patient support and each of the PST sessions participants will be offered food and drink.

#### Poroporoaki - Farewell

Poroporoaki, or speeches of farewell, are reserved for the final part of the powhiri but are by no means the least important part of the process. The poroporoaki is the act of farewell and the return of mana (esteem and authority) to the host people. This is operationalised by the writing and sharing of a final discharge letter to other people involved in the participants care.

#### Intervention group

Māori randomised to the experimental group (Te Ira Tangata) who give their consent will be offered the following by the self harm team:

1. Patient support for up to two weeks. This will consist of one or two face-to-face or telephone sessions depending on patient preference and feasibility over the two week period following the participant's discharge from hospital. These sessions will involve obtaining the discharge plan developed by the assessing clinicians, checking that the patient understands it, identifying potential barriers to implementation of the plan and assisting the patient to follow through with the plan. In other words, the primary aim of patient support will be to ensure patients do not "fall through the cracks". The research clinicians will be expected to liaise with the mental health crisis and community mental health teams; alcohol and drug services; primary care and non health services. Each patient support session will include a risk assessment asking about thoughts and plans for self harm. If a patient is identified as being at risk of self harm the risk management protocol will be followed.

2. Postcard contact for one year. Eight postcards will be sent in sealed envelopes in months 1,2,3,4,6,8,10 and 12 after the index episode. The cards will contain a short message stating that we hope things are going well and inviting them to write us a note if they wish to (Additional file 2). Each envelope will contain a return stamped addressed envelope.

3. Problem solving therapy. This will consist of four to six sessions in the four weeks after the participant's index presentation to hospital for self harm. Research clinicians will assess the participant's eligibility for brief problem solving therapy prior to and at the initial patient support session. Patients may be ineligible for brief PST if they are already receiving psychotherapy (for example if they are receiving Dialectical Behaviour Therapy), if brief PST would contradict their management plan, if they live or are moving out of area, if they are in prison or if there is a risk of harm to the research clinician. The problem solving therapy we will use in the treatment package will be conducted with individual patients and is based on the model originally defined by D'Zurilla and Goldfried [21]. PST sessions will aim to teach the person to recognise and identify current problems and will provide them with a structured approach to problem solving. A clinician manual and a participant workbook will be used by the research team to guide the structure of PST sessions. Sessions will be audiotaped.

4. Improved access to primary care. We will encourage participants to attend their GP for a physical health check paying particular attention to cardiovascular risk factors especially alcohol and smoking. We will use GP vouchers to facilitate these visits.

5. A risk management strategy. The teams will also pilot a risk management strategy around the management of suicidal patients. This will consist of a checklist for patient support to ensure that key tasks are completed and questions asked. Secondly the research team will meet once a week to discuss adverse events defined as repeat episodes of self harm, hospital re presentation for any reason and suicides. A record will be kept of these discussions, including any changes to process as a result of these discussions, and circulated to the team in the form of a "risk bulletin". The research team will also receive training in crew resource management.

6. Cultural assessment. We have two aims here, the first is to increase the number of people who receive cultural services after self harm. From our previous study we found that the input of Maori services after self harm was very rare. The second aim will be to complete a cultural assessment on everyone paying particular attention to the sense of belonging and feelings around ethnicity. Problems with sense of belonging will be included in the problem solving checklist for patients.

#### Control group

Māori patients randomised to the control group who give their consent will receive treatment as usual and will be asked to complete a consent form and questionnaires at baseline, and questionnaires and telephone interviews at three months and one year. Treatment as usual following self harm varies and may involve referrals to multi-disciplinary teams for psychiatric or psychological intervention, referrals to crisis teams and/or recommendations for engagement with community alcohol and drug treatment centres. The discharge plan may include referrals to more than one health care provider, or may consist solely of referral back to the patient's General Practitioner.

##### Treatment as usual assessment

Treatment as usual for all participants will be assessed by self report using a written questionnaire and telephone interview by a research assistant blind to treatment allocation; a review of DHB records; and by using the National minimum dataset from the Ministry of Health Information Directory to record hospital contacts and contact with mental health services.

#### Outcome measures (Table 1)

**Table 1:** Outcome measures

Outcome measure	Description	Explanation	Administered
**Primary**			

Hopelessness	Beck Hopelessness Scale (BHS)[22]	Scores below 9 on the BHS. Best predictor of subsequent self harm. Scores on a range of 0 to 20 with higher scores indicating greater hopelessness.	Entry, three and twelve months

**Secondary**			

Hospital repetition of self harm		Data on hospital contacts from participating DHB's and the New Zealand Health Information Service National Minimum Dataset	Three and twelve months

Depression and anxiety	Hospital Anxiety and Depression Scale (HADS)[23]	Self report scale. Scores of 10 and above on the anxiety and depression sub scales indicate clinically significant symptoms.	Baseline, three and twelve months.

Health status	EQ-5D [24]	A generic health-related quality of life index that can be related to costs.	Baseline, three and twelve months.

Self report repetition of self-harm		Self report assessed by telephone interviewer blind to allocation	Three and twelve months

Social functioning	SF-36 [25]	A generic measure of functional health and well being	Baseline, three and twelve months

Sense of belonging	Sense of belonging instrument (SOBI) [26]	Self report scale on sense of belonging to a community and ethnicity	Baseline, three and twelve months

Cultural identity	Cultural identity profile [27]	Produces four indicators of cultural identity, a secure, positive, notional or compromised identity	Baseline, three and twelve months

Seriousness of suicide attempt	Self rated objective part of the Beck Suicide Intent Scale (BSIS)[28]	Self report scale indicating the degree of suicidal intent of the self harm episode	Baseline

Costs following index attempt	Health service use, costs of attending care, cost of medication and time off work	Self report assessed by telephone interviewer blind to allocation	Three and twelve months

##### Intervention study

The primary outcome measure is the proportion of subjects who score below nine on the Beck Hopelessness Scale after one year (see sample size section for explanation).

Secondary outcomes are:

1. The proportion of subjects who repeat self harm by presenting to hospital at three months and one year after their index attempt.

2. Self reported repetition of self harm at three months and one year, assessed with a telephone questionnaire and a written questionnaire

3. Hopelessness measured by the Beck Hopelessness Scale at baseline, three months and one year

4. Anxiety and depression measured by the Hospital Anxiety and Depression Scale at baseline, three months and one year

5. Quality of life as measured by the EQ-5D (http://www.euroqol.org/) and the SF36 at baseline, three months and one year

6. Overall mortality (that is suicide deaths plus other causes of death) and suicide deaths at three months, one year, five years and ten years

7. Health service use at three months, one year, five years and ten years, assessed by a telephone questionnaire and interrogation of DHB and Ministry Of Health Information Directorate records

Outcome measures will be collected in several different ways.

##### Self report or self rating

Ratings scales will be by self rating. Self report will be to a researcher blind to the treatment allocation by a semi structured telephone interview which asks about repetition, health service use and economic measures (see attached copy of questionnaire).

##### DHB records

we will inspect these for episodes of repetition and health service use.

##### Ministry Of Health Information Directorate records

we will inspect these for health service use (both general and mental health), repetition and for mortality data. (It is necessary to look at national data on these measures as we found in our previous trial that at least 50% of people who self harm change address over twelve months often outside the DHB where they presented).

###### Self report by structured interview

Information for the economic analysis and self report of repetition will be gathered by telephone interview at three months and one year after the index attempt (Additional file 3). Telephone interviewers will be blind to the allocation of subjects. Blinding will be tested by asking the interviewers to nominate which group the subject was enrolled in. The economic analysis will also use a brief measure of quality of life the EQ-5D and interviewers will ask about health service use; costs associated with this; time off work; time off work for family to care for the participant; changes in benefit; changes in occupation; and drug use and cost. Participants will have the option of completing these measures in a face to face interview if, for example, they do not have a phone.

#### Process evaluation

A process evaluation will explore the implementation, receipt and context of the intervention with a view to helping understand the results in accordance with the Medical Research Council's guidelines [29] on assessing complex interventions. This will describe the processes in the intervention and control groups, provide information about the contexts in which the treatments are delivered and supply information about the experience of being part of the trial. The process evaluation is described in Table 2 below. The self-harm teams will also receive weekly supervision, the main themes of which we will incorporate into the process evaluation.

**Table 2:** Process evaluation in Te Ira Tangata

Data collection method	Data collected
Programme documentation and observation (to assess fidelity, dose and reach)	Number of sessions of patient support Number of PST sessions Completion of PST Audiotaping of PST sessions Examination of written client PST research records Number of clients where sense of belonging addressed in PST Use of GP voucher Number of postcards sent Summary of discussions around adverse events Proportion of clients who received the interventions in each centre Proportion of clients not contactable after presentation to hospital
Structured interviews (to assess barriers, facilitators and suggestions for improvement)	Interview research clinicians re barriers and facilitators to the interventions plus suggestions for improvement Interview purposive sample of patients re what helped and what did not help plus suggestions for improvement Twelve month telephone interview of all patients what helped and what did not help Interview GP's who saw clients through GP voucher to assess their perception of the intervention and suggestions for improvement Interview focus group of staff in mental health and cultural services re barriers and facilitators to interventions plus suggestions for improvement

#### Process evaluation analysis

Numerical data will be entered into the Clinical Trials Research Unit web based data entry system specifically designed for this study. Information from the examination of patient notes and audiotapes will be used to assess the adherence of therapists to the manual in a 10% random sample of those who completed problem solving therapy. Data from structured interviews and focus groups will be analysed for emergent themes using NVIVO.

### Statistical methods

#### Power analysis

We know from the previous trial that approximately 310 Māori present with self harm each year to the general hospitals in Waitemata, Counties Manukau and Northland DHB's [30]. Of these 70% or 217 would have been eligible for this study. Assuming that we recruit participants at the same rate as the previous trial, that is 50% of eligible people, we would be able to recruit about 105 people a year from these DHB's, (or about 150 over eighteen months).

Traditionally the main outcome measure in self harm intervention research has been the proportion of people repeating after one year which is generally around 18% (29). However there are two problems here one of which is particularly relevant for indigenous people. Firstly to significantly reduce this by a third to 12% would need nearly 600 people in each arm of a trial (assuming a power of 80% with a significance level of 0.05). This is particularly problematic in trials involving indigenous people who are less numerous than the majority population. In New Zealand there are about 1000 episodes of self harm involving Māori each year which represents about 840 people. Assuming similar rates of eligibility and recruitment as in the earlier trial this means that the maximum number of Māori who could be enrolled in a treatment trial powered on a significant reduction in the repetition rate is about 300 people a year. In other words a trial would have to involve every DHB in the country and recruit people for four years. We suspect that the chances of funding any such trial are slim. The second problem with repetition rates is that although they are clearly a key consideration they are not important for the four out of five people who do not repeat. Other outcome measures such as measures of distress, quality of life and cultural identity are also significant. For these reasons we have decided that the main outcome we are interested in after one year is the proportion of people scoring below 9 on the Beck Hopelessness Scale. We have chosen this measure for several reasons, it is the best predictor of future self harm attempts; it is a significant marker of distress; and we know from previous research that individuals who score 9 or more on this measure are at least ten times more likely to kill themselves in the next year. Lastly in our previous trial we had outcome data at three months or one year on this scale in 85% of cases. In the previous ACC trial the proportion scoring 9 or more in both groups at baseline was 50%. Using this as the main outcome measure with 80% power, a significance level of 0.05 and a reduction in the proportion scoring above 9 from 50% to 25% after a year will require 66 people in each group, a total of 132. Allowing for 15% non completion of the Beck Hopelessness Scale we will need to recruit 155 people which should be feasible to collect over the fifteen months of recruitment into this trial. The figure of 155 participants is those people who have been randomised and consented to be in the study. We would expect that we would need to randomise about double this number of people to achieve this target.

### Analysis

#### Statistical analysis will be by the biostatistics team of the Auckland Clinical Trials Research Unit (CTRU)

Data from the trial will be entered into an Oracle database at the CTRU and extracted into SAS for analysis. All statistical analyses will be performed using SAS version 9.2 (SAS Institute Inc. Cary NC). All statistical tests will be two-tailed and a 5% significance level maintained throughout the analyses. Assessment of baseline comparability of the intervention and control group will be carried out via descriptive analyses for demographic information, method of self-harm, and previous history of self-harm. The proportion of people repeating self-harm in each group will be analysed using chi-squared test. We plan to analyse people whose index episode is a first presentation and those whose index is a repeat separately and together. The number of self-harm re-presentation episodes for the hospital and self report outcomes during follow-up will be analysed using negative binomial regression. Kaplan-Meier curves and Cox proportional hazards regression modeling will be used to analyze time to first re-presentation to hospital for self-harm and time to event for the mortality outcomes. The change from baseline to 3 months and one year in each of the repeated continuous outcomes will be analysed using mixed model regression. If baseline characteristics are found to be substantially different between the groups we will adjust for these in the regression modeling. All analyses will be conducted on patients who are randomised and consented except for the re-presentation to hospital and time to re-presentation outcomes which will be an intention to treat analysis that includes all randomised patients including those that did not consent.

In addition sensitivity analyses will be conducted using a CACE analysis [31] where appropriate which takes into account the fact that after randomisation not everyone in a Zelen design agrees to take part in the study. This has the effect of diluting any treatment effect and introducing a possible self selection bias. A CACE analysis is an attempt to correct for this. The assumption behind this is that those who consent to the intervention and treatment as usual are similar. We will test this by comparing the main factors which affect outcomes in both groups including the proportion of people presenting for the first time, gender and age.

### Cost effectiveness analysis

We aim to collect the following data from all patients in the trial at three months and one year after the date of their index attempt. The data will be collected by a research assistant by telephone interview with the patients, by examination of routinely collected health data and liaison with the finance departments of the relevant health care providers.

#### Costs to patients

• Time off work

• Distance travelled for treatment for all disorders

• Time taken for treatment

• Costs for attending general practice - travel, payment to general practice, time off work

• Costs of family to attend treatment or provide support for the patient (for example taking time off work to be with the patient)

• Cost of medication

• Benefits claimed

#### Costs to health care provider

• Staff salaries for providing treatment including the problem solving therapy (therapist and in-patient treatment)

• Length of stay in hospital

• Cost of treatments - for example care in intensive care or burns unit, cost of medication

• Overheads

##### Analysis

All analyses will be carried out on an intention-to-treat basis for total costs over three months and one year. We will perform multiple regression to adjust for baseline characteristics including age, sex, number of previous attempts and Beck Hopelessness Score. We intend to perform an incremental analysis of costs and consequences using the primary outcome measure as the number of repetitions averted. From this we will produce cost-effectiveness acceptability curves for the intervention.

We also intend to perform a sensitivity analysis to test how the costs and consequences of self-harm change within a range of costs for the different economic inputs. We anticipate that the model will be most sensitive to changes in the costs of in-patient medical care. We will also test the sensitivity of the results to productivity losses and costs of mental health treatment.

###### Termination of the study

Termination will be considered if there is 10% absolute greater number of adverse events (re-presentations to hospital for self harm) in the treatment group than in the usual care group at three months. Unblinded analyses to assess excess harm will be conducted at one year by an independent statistician at CTRU (not the study statistician). One year was chosen as any shorter time will mean that there will be few outcomes and any longer time is close to the end of the intervention at 18 months. For this analysis the Haybittle-Peto stopping boundary will be used which is based on a three standard deviation rule corresponding to a two sided test P = 0.003 stopping rule. This does not affect the power calculation of the study.

Ethical approval has been received from the New Zealand, Central Health Ethics Committee.

## Discussion

The study, due to report its findings in 2012, tests the effectiveness of a complex package of interventions in the management of Maori who present to hospital with intentional self-harm. It uses a novel design to try and overcome the problems of previous trials which have recruited small numbers of unrepresentative people. A strength of the study is that it is a pragmatic trial which aims to demonstrate the importance of a comprehensive approach to culture in assessment and treatment. A potential limitation is the analysis of the results which is complex and may underestimate any effect on the secondary outcomes if a large number of people refuse their consent in the group randomised to problem solving therapy as they will effectively cross over to the treatment as usual group. Another potential issue is collecting a high enough proportion of completed rating scales at follow up.

## Abbreviations

BHS: Beck Hopelessness Scale; BSIS: Beck Suicide Intent Scale; CACE: Complier Average Causal Effect; CTRU: Clinical Trials Research Unit; DHB: District Health Board; GP: General Practitioner; HADS: Hospital Anxiety and Depression Scale; PST: Problem solving therapy; SF36: Short Form (36) Health Survey; SOBI: Sense of belonging instrument

## Competing interests

The authors declare that they have no competing interests.

## Authors' contributions

All authors contributed to the study design and study protocol. SH is the principle investigator, NC is the project manager and KW is the project co-ordinator. MD, HE, RT are co-investigators. SH and NC drafted the article. All authors have read and approved the final manuscript.

## Supplementary Material

Additional file 1 **EDcard**. A copy of the card given to potential participants informing them that they can refuse to be approached about the study. Click here for file

Additional file 2 **TIT WDHB**. A copy of the postcard given to consenting participants in the intervention arm. Click here for file

Additional file 3 **TIT3 Month Telephone Interview**. The telephone interview of all consenting participants at three months Click here for file
