# Supplementary material for: Te Ira Tangata: A Zelen randomised controlled trial of a treatment package including problem solving therapy compared to treatment as usual in Maori who present to hospital after self harm
Source: Trials. 2011 May 11;12:117. doi: 10.1186/1745-6215-12-117 (PMC3103449; doi:10.1186/1745-6215-12-117)
Supplement: Additional file 1 — EDcard. A copy of the card given to potential participants informing them that they can refuse to be approached about the study. [file 1745-6215-12-117-S1.PDF]

Te Ira Tangata study

In order to improve services for Maori who present to hospital following self harm, it is important that we know what treatment Maori are offered, what they receive and what treatment works.

Health professionals and researchers at the University of Auckland are currently working with staff and patients at North Shore, Waitakere, Middlemore and Whangarei hospitals on a study that aims to answer these questions.

The research team would like to contact you while you are in hospital or soon after your discharge from hospital to explain the study in detail and see if you would like to take part. The conversation should take no more than 5 or 10 minutes. You do not have to take part, and if you do refuse this will in no way affect the care you receive.

If you do not wish to be contacted at all please tell the person who gave you this card or email: teiratangata@auckland.ac.nz

THE UNIVERSITY OF AUCKLAND
NEW ZEALAND
Te Whare Wānanga o Tāmaki Makaurau
