# Supplementary material for: Te Ira Tangata: A Zelen randomised controlled trial of a treatment package including problem solving therapy compared to treatment as usual in Maori who present to hospital after self harm
Source: Trials. 2011 May 11;12:117. doi: 10.1186/1745-6215-12-117 (PMC3103449; doi:10.1186/1745-6215-12-117)
Supplement: Additional file 2 — TIT WDHB. A copy of the postcard given to consenting participants in the intervention arm. [file 1745-6215-12-117-S2.PDF]

Waitemata
District Health Board
Te Wai Awhina

Liaison Psychiatry
Te Ira Tangata Study
North Shore Hospital
Shakespeare Road
Takapuna
Private Bag 93503
Takapuna
Auckland

Tena Koe

It has been a short time since you were in hospital,
and we hope things are going well for you.

If you wish to drop us a note we would be happy to hear from you.

Best Wishes
Dr Simon Hatcher

THE UNIVERSITY OF AUCKLAND
FACULTY OF MEDICAL AND HEALTH SCIENCES

teiratangata@auckland.ac.nz

Te Ira Tangata

Waitemata
District Health Board
Te Wai Awhina

Liaison Psychiatry
Te Ira Tangata Study
North Shore Hospital
Shakespeare Road
Takapuna
Private Bag 93503
Takapuna
Auckland

Tena Koe

It has been a short time since you were in hospital,
and we hope things are going well for you.

If you wish to drop us a note we would be happy to hear from you.

Best Wishes
Dr Simon Hatcher

THE UNIVERSITY OF AUCKLAND
FACULTY OF MEDICAL AND HEALTH SCIENCES

teiratangata@auckland.ac.nz

Te Ira Tangata

Tena koe,

E ahua poto te wa i kite matou i a koe ki roto i te hohipera,

It has been a short time since you were in Hospital,

Te tumanako kei te tino pai to ora

and we hope things are going well for you.

Mehemea e hiahia ana koe te tuku panui mai, e hari koa ana matou!

If you wish to drop us a note we would be happy to hear from you.

Nga mihi

Best wishes

Dr Simon Hatcher

Waitemata District Health Board

Te Wai Awhina

THE UNIVERSITY OF AUCKLAND

FACULTY OF MEDICAL AND HEALTH SCIENCES

He aha te mea nui o tenei ao?
He tangata, he tangata, he tangata.

What is the greatest thing in the world?
It is people, it is people, it is people

Te Ira Tangata

Tena koe,

E ahua poto te wa i kite matou i a koe ki roto i te hohipera,

It has been a short time since you were in Hospital,

Te tumanako kei te tino pai to ora

and we hope things are going well for you.

Mehemea e hiahia ana koe te tuku panui mai, e hari koa ana matou!

If you wish to drop us a note we would be happy to hear from you.

Nga mihi

Best wishes

Dr Simon Hatcher

Waitemata District Health Board

Te Wai Awhina

THE UNIVERSITY OF AUCKLAND

FACULTY OF MEDICAL AND HEALTH SCIENCES

He aha te mea nui o tenei ao?
He tangata, he tangata, he tangata.

What is the greatest thing in the world?
It is people, it is people, it is people

Te Ira Tangata
