# Supplementary material for: Te Ira Tangata: A Zelen randomised controlled trial of a treatment package including problem solving therapy compared to treatment as usual in Maori who present to hospital after self harm
Source: Trials. 2011 May 11;12:117. doi: 10.1186/1745-6215-12-117 (PMC3103449; doi:10.1186/1745-6215-12-117)
Supplement: Additional file 3 — TIT3 Month Telephone Interview. The telephone interview of all consenting participants at three months [file 1745-6215-12-117-S3.DOC]

**Index date:**

**Study Number: NHI:**

**Name:**

**Address:**

**Home Ph:**

**Mobile Ph:**

__________________________________________________________________________________________________

**GP: Phone:**

**__________________________________________________________________________________________________**

**Alt Contact (1):**

**Name:**

**Phone:**

__________________________________________________________________________________________________

**Alt Contact (2):**

**Name:**

**Phone:**

| **NOTES:** | | | |
| --- | --- | --- | --- |
| **Introduction**  Kia ora, my name is _____________ and I am with the Te Ira Tangata Study ( University of Auckland).  Did you receive the questionnaires I posted to you recently? Do you have any questions regarding these?  As part of the Study we are inviting people to answer a telephone questionnaire that will take about 10 minutes. Would you be happy to complete this questionnaire now or at a more time suitable to you? You are not required to answer all of the questions. You can stop the interview at any time and this information is confidential to the Study. | | |  |
| 1. **Have you had contact with one of the following since you agreed to participate in this study? If yes, please specify number of contacts:**   A staff member from the Community Mental Health Team  A staff member from the Alcohol & Drug Team  A Private Psychiatrist, Psychologist or Counsellor  Maori mental health  Pacific Island Cultural Support  Asian Cultural Supoprt  Tohunga (Maori Healer)  Kaumatua (Elder)  Whanau  Marae (Village Common)  Hahi Maori (Maori Church)  Iwi Authorities  Runaka/ Runanga  Maori Health Services  Maori NGO  Hui | **FACE to FACE** | **TELEPHONE** |  |
| 1. **If you attended a treatment session with one of the above, did you take a family/whanau member or friend with you?** | **Yes or No**  If yes, how many sessions did they attend? ____ | |  |
| 1. **Did you have to pay for child care to enable you to attend any appointments?** | **Yes/No**  If yes, how much did it cost in total $______ | |  |
| 1. **Did you have to pay for travel to these appointments?** | **Yes/No**  If yes, please estimate total cost $_______ | |  |
| 1. **Are you currently on any psychiatric medication?** | **Yes/No**  If yes,  Name of medication: ______________________  How long have you been taking that? ___________  Name of medication: ______________________  How long have you been taking that? ­­­­­­­__________  What was the cost of your last prescription for this medication? $_______ | |  |
| 1. **Are you currently working** | **Yes/No/Studying**  If yes, how many hours a week are you employed ____ | |  |
| 1. **What is your annual income before tax? (this is so we can do an economic analysis of the Study)** | **0 - $20,000**  **$20,000 - $40,000**  **$40,000 - $60,000**  **$60,000 - $80,000**  **$80,000 - $100,000**  **Over $100,000** | |  |
| 1. **Since you agreed to participate in this Study , have you had to take any time off work to attend health care appointments?** | **Yes/No**  If yes, how many hours? ____ | |  |
| 1. **Since you agreed to participate in this Study, have you had to take any sick time off work?** | **Yes/No**  If yes, how many days in total? ____ | |  |
| 1. **Has anyone taken time off work to look after you since you agreed to participate in this Study?** | **Yes/No**  If yes, for how many days? ____ | |  |
| 1. **Have you harmed yourself again since you agreed to participate in this Study?** | **Yes/No**  If yes, how many times? ____  For each time specify method:  OD ____  CO Poisoning ____  Ingestion of toxic substance ____  Hanging ____  Cutting ____  Jumping from a height ____  Other ____ | |  |
| 1. **As a result of one or more of these episodes of self harm did you go to hospital?** | **Yes/No**  If yes, how many times? ____  In total how long did you spend in hospital? ____ days | |  |
| 1. **Have you attended your general practice since you agreed to participate in this Study?** | **Yes/No**  If yes, how many times? ____  How much does a GP visit usually cost you? $____ | |  |
| 1. **Have you been admitted to hospital for any reason not related to self harm since you agreed to participate in this Study?** | **Yes/No**  If yes, was the admission for one of the following please note how many admissions for each:  Medical/Surgical ___  Psychiatric ___  Obstetric ___ | |  |
| 1. **Are you on a Benefit?** | **Yes/No**  If yes, which one?  **Unemployment , DPB, Sickness, Invalid’s, ACC, Superannuation**  Has this changed since we last contacted you? **Yes/No**  If yes, have you started claiming a benefit since your presentation?  **Yes/No**  Have you stopped claiming a benefit since your presentation? **Yes/No**  Have you changed then benefit you claim? **Yes/No** | |  |
| 1. **Were you born in NZ?** | **Yes/No**  **If No, how long have you lived in NZ?**  **_____**weeks/months/years | |  |
| 1. **Have you moved house or changed where you live in the last 12 weeks?** | **Yes/No**  If yes, go to question 18 | |  |
| 1. **Update contact details** |  | |  |

We will be sending you some questionnaires to complete in the post. When you return these in the SAE, we will send you a $60 grocery voucher. Thank you for your time.
